# Study protocol: follow-up home visits with nutrition: a randomised controlled trial

**DOI:** 10.1186/1471-2318-11-90

**Published:** 2011-12-28

**Authors:** Anne Marie Beck, Stine Kjær, Birthe S Hansen, Rikke L Storm, Kirsten Thal-Jantzen

**Affiliations:** 1 EFFECT, University Hospital Herlev, Herlev Ringvej, DK-2730 Herlev, Denmark; 2 Department of Human of Nutrition, Faculty of Life Sciences, University of Copenhagen Rolighedsvej 30, DK-1958 Frederiksberg C, Denmark

## Abstract

**Background:**

Geriatric patients are at high risk of re-admission after discharge. Pre-existing nutritional risk amongst these patients is of primary concern, with former nutritional intervention studies being largely ineffective. None of these studies has included individual dietary counselling by a registered dietician or has considered competing medical conditions in the participants. A former randomised study has shown that comprehensive discharge follow-up in geriatric patients homes by general practitioners and district nurses was effective in reducing the re-admission risk in the intervention group compared to the control group. That study did not include a nutritional intervention. The purpose of this study is to assess the combined benefits of an intervention consisting of discharge follow-up in geriatric patients' home by a general practitioner and a registered dietician.

**Methods/design:**

This single-blind randomised controlled study, will recruit 160 hospitalised geriatric medical patients (65+ y) at nutritional risk. Participants will be randomly allocated to receive in their homes, either 12 weeks individualised nutritional counselling by a registered dietician complemented with follow-up by general practitioners or a 12 weeks follow-up by general practitioners alone.

**Discussion:**

This trial is the first of its kind to provide individual nutritional intervention combined with follow-up by general practitioner as an intervention to reduce risk of re-admission after discharge among geriatric medical patients. The results will hopefully help to guide the development of more effective rehabilitation programs following hospital admissions, which may ultimately lead to reduced health care costs, and improvement in mobility, independence and quality of life for geriatric patients at nutritional risk.

**Trial Registration:**

ClinicalTrials.gov 2010 NCT01249716

## Background

Undernutrition is common in old people admitted to the hospital and nutritional state often deteriorates further during hospital stay [1]. Therefore, at discharge a high amount of old patients will still be undernourished or at-nutritional risk: A recent study among 2076 old rehabilitation patients (80.6 y) have found that 85% were at risk of undernutrition (MNA 17-23.5) or undernourished (MNA < 17) according to the Mini Nutritional Assessment (MNA) and that length of stay was higher in those two groups compared with the well-nourished (p < 0.001) by 18.5 and 12.4 days respectively [2]. And an older study among old people (81 y) discharged to their own home has found that those with empty refrigerators were more frequently readmitted and three times sooner than those who did not have empty refrigerators [3].

Potentially the period after discharge is the most important time to intervene, because hospital stays are generally short and getting shorter.

Further, according to the Resolution from the Council of Europe patients in need of nutritional support should receive such treatment before admission (where possible), at the earliest opportunity during hospital stay and after discharge [4].

In spite of this, there is a dearth of published evidence of benefit or harm:

According to our knowledge six studies has assessed the benefits of oral nutritional support to geriatric patients at risk of or already undernourished, initiated in relation to discharge [5-11].

In most studies a positive effect of the intervention was found on the energy and nutrient intake, the nutritional status and in some also the functional status. In contrast the effect on the rehabilitation capacity, the quality of life and the survival was very limited.

One explanation for the limited effect observed could be that the length of the majority of studies was relatively short, 4-8 weeks. This may be a problem since e.g. it can be seen from the data presented by Miller and co-workers [10] that participants in both intervention and control group continues to loose weight, both during the 6 weeks intervention and in the 6 weeks after.

Another explanation for the limited effect could be the high number of re-admissions - especially found in the studies among the medical patients. This may have worsened the outcome for an already very frail population. Inappropriate medical treatment often has inadvertent effects, and a considerable number of admissions are attributable to inappropriate medical treatment that could be avoided. In a former randomised Danish study it was shown that comprehensive discharge follow-up in geriatric patients homes by general practitioners (GPs) and district nurses was reducing the re-admission risk in the intervention group compared to the control group after 12 weeks (29 vs. 39%, p = 0.044) [12]. The main focus in that study was GPs follow-up on hospital treatment, and medications, with no special emphasis on nutrition.

A third explanation for the limited effect could be the relatively low level of compliance with the oral nutritional supplements reported in some of the earlier studies [5,6,9]. None of these studies have included individual goal setting, energy dense menus, and counselling focussing on nutritional risk factors, i.e. the expertise from a registered dietician (RD).

All in all, a comprehensive approach to nutrition support, rather than commercial oral nutritional supplements alone, is likely to be required to improve nutritional status and prevent re-admissions, and hence impact positively on functional outcomes and quality of life.

The purpose of this study is to assess the combined benefits of an intervention consisting of discharge follow-up in geriatric patients' home by a GP and a RD.

## Methods/design

### Design

This study is designed as a randomised controlled trial comparing discharge follow-up in patients' home by GP vs. discharge follow-up in patients' home by GP and RD. Patients are eligible for this study when they are 65+ years old and at nutritional risk according to the level 1 screen in NRS2002 [13].

The primary outcome parameter will be the prevalence of re-admissions in the intervention and control group. Secondary outcomes will be changes in body weight, muscle strength, quality of life, and rehabilitation capacity.

### Feasibility of recruitment and sample size

An earlier study has shown that 50% of the elderly hospital population is at nutritional risk according to the level 1 screen [14]. For a clinically relevant difference of 10% in re-admissions and an expected drop-out rate of 12% (based on [12]), a statistical significant level of 0.05 and a power of 80%, two groups of 80 patients are calculated to be necessary.

A pilot study has shown that inclusion of up to 10 patients at nutritional risk per week is feasible. Taking in account an expected refusal rate of 30% at inclusion and loss to follow-up of 10% during the 12 weeks intervention, we aim to include two groups of 90, to be reached in approximately 6 months.

### Randomisation

Patients will be randomised after discharge, right before the baseline assessment. Participants, the GPs and RDs (RLS and KT-J), the principal investigator (AMB) and research assistants (BSH, SK) are not blinded for the intervention. Before starting the analysis the principal investigator will be re-blinded for patients' group assignment.

### Population, inclusion and exclusion criteria

All elderly patients (65+ years of age, living in three municipalities (Herlev, Rødovre or Gladsaxe)), hospitalised for minimum two days at the wards of geriatric medicine of the University Hospital of Herlev, will be screened by a research assistant for nutritional risk. Patients will be excluded from the study when they; suffer from senile dementia or terminal disease; can not understand the Danish language; are residing in nursing homes; or are not able to or willing to give informed consent.

### Nutritional status

Patients are eligible for this study if they are identified at nutritional risk according to the following criteria in the level 1 screen NRS2002 [13]:

▪ Is Body Mass Index (BMI in kg/m^2^) < 20.5? and/or

▪ Has the patient lost weight within the last 3 months? and/or

▪ Has the patient had a reduced dietary intake in the last week? and/or

▪ Is the patient serious ill? (e.g. in intensive therapy)

▪ The nutritional risk will be confirmed by the research assistant by means of medical records.

### Intervention

#### Discharge follow-up in all patients' home by GPs

The follow-up consists of three contacts, conducted approximately one, three and eight weeks after discharge in both control and intervention patients. The contacts are guided by an agenda (based on [12]):

▪ Checking the discharge letter for specific recommended paraclinical or clinical follow-up

▪ Check need for adjustment of medication

▪ Check of the family's medical cabinet

▪ Checking the general health status (nutrition (vitamin D), physical activity, alcohol, continence, depression, dementia, and so on)

The contacts are either in the GPs clinic or as a home visit depending on the patients overall condition.

#### Patients randomised to nutritional intervention

The research registered dieticians (RLS and KT-J) will perform a comprehensive nutritional assessment at the first home visit, as a basis for developing a nutrition care plan consistent with estimated nutritional requirements and nutritional rehabilitation goals. Basal metabolic rate will be assessed by means of Harris-Benedict and a factorial method, eventual accounting for weight gain factors, will be used to estimate the total energy- and protein requirement for each patient (based on [15]).

To assess dietary intake, the RDs will perform a standardised dietary interview with each participants to determine total energy and protein intake at each visits. Strategies for achieving energy and protein requirements will include dietary counselling with attention to nutritional risk factors, timing, size and frequency of meals, recommendations for nutrient dense foods and drinks, and provision of leaflets with information. Supplementation with energy and protein-dense meals-on-wheels, subscription of commercial oral nutritional supplements as well as vitamin D, calcium and other vitamin-minerals will also be considered to achieve optimal nutritional status.

All in all, the RDs will perform three home visits, to perform dietetic care and maximise participants' nutritional status by way of reviewing the nutrition care plan, dietary counselling, motivation and education, monitoring participant weight, and ensuring energy and protein requirements are achieved. If it is considered relevant the participants will receive short follow-up consultations by telephone by the RDs in order to give advice and to stimulate compliance to the proposed nutritional intake (in-between the home visits).

At least one counselling will be together with the patient's GP, in order to discuss the treatment, either in home or at the GPs clinic.

### Procedure

After obtaining patients informed consent (either at the hospital or right after discharge) an inventory will be made of possible confounders. This includes the following characteristics:

▪ Socio-demographic data (age, gender)

▪ Medical diagnosis

▪ New Mobility Score (assessment of mobility before admission (total score 0-9) [16]

▪ Additional discharge interventions (e.g. outgoing hospital teams, discharge follow-up phone calls etc.)

▪ Prescription/use of commercial oral nutritional supplements

▪ Prescription of Vitamin D supplements

▪ Prescription of rehabilitation in the form of physiotherapy

After 12 weeks participants will be contacted via telephone and mail to organise the follow-up assessment.

If there is no response then the research assistants will contact the hospital to check for an eventual re-admission.

### Outcome parameters

Outcome parameters will be measured in the participants' home as soon as possible after discharge (t = 0) and at +12 weeks (t = 12).

Primary outcome is prevalence of re-admissions. All outcome parameters that will be measured are listed below. If nothing else is stated the data is gathered by the research assistants.

#### Re-admissions (t = 12)

A register-based evaluation of readmissions will be done after 12 weeks. Data on admission to the hospital will be based on the National Patient Register. Information about the number of days to first re-admission and the number of days spent in hospital will also be collected from the Register.

#### Nutritional status (weight, height, BMI) (t = 0 and t = 12)

Weight is measured (with patients wearing light indoor clothes and no shoes). Information about weight will also be obtained by the RDs during the visits to the intervention group.

BMI is calculated as actual weight in kilograms divided by the square of height in meters.

As measurement of height is often not feasible in this chronic diseased, old and frail population, data on height will be retrieved from self-reported height.

#### Dietary intake (t = 0 and t = 12)

Dietary intake will be assessed by means of a 4-days dietary record. Participants will receive instructions from the research assistants on how to fill in the dietary record. They will receive the dietary records in advance of the visits at t = 0 and t = 12. At the visits the finalised records will be inspected and ambiguous entries clarified. The intake of energy and nutrients will be calculated by means of a computer program based on the Danish food composition table (available at: http://www.foodcomp.dk).

#### Hand grip strength (t = 0 and t = 12)

Hand grip strength (in kg) will be measured with a Jamar 5030J1 Hydraulic Hand Dynanometer. Participants will be seated with forearms rested on the arms of the chair. They are asked to perform three maximum force trials with their dominant hand and using the second handle position. The maximal grip score from the three values will be used.

#### Chair stand (t = 0 and t = 12)

To test the physical performance, the participants are asked to fold their arms across their chest and to stand up and sit dawn on a chair without pushing off with arms, as many times as possible during 30 seconds. The arms may be used for assistance or for safety if need [17].

#### Cognitive performance (t = 0 and t = 12)

The Mini Mental State Examination (MMSE) will be administered to assess cognitive status of the participants. The MMSE is a widely used and easily administered test of cognitive status. It consists of 11 tasks and is graded to assign old people a score in the range of 0 to 30.

Participants, who has difficulties with seeing, hearing or writing, will not be asked to complete the MMSE-test.

#### Activities of Daily Living (t = 0 and t = 12)

The ability to participate in activities of daily living (ADL) will be assessed using the validated de Morton Mobility Index (DEMMI) [18]. The DEMMI is a 15-item one-dimensional instrument that measures mobility across the spectrum from bed bound to independent mobility. The raw score total (0-19) must be converted to a DEMMI SCORE (0-100 where 100 is independent mobility).

#### Disability and tiredness in daily activities (t = 0 and t = 12)

Disability is measured by a validated scale (the Mob-H Scale) by asking questions about need of help in the following six activities: (1) transfer, (2) walk indoors, (3) get outdoors, (4) walk out of doors in nice weather, (5) walk out of doors in poor weather, and (6) manage stairs.

Tiredness in daily activities is measured by asking the participants if they feel tired after performing the same six activities [19].

#### Health-related quality of life (t = 0 and t = 12)

Quality of life is measured by questions from SF-36 regarding physical functioning; role-physical; bodily pain; general health; vitality; social functioning; role-emotional; mental health and health transition http://www.sf-36.org.

#### Rehabilitation capacity (t = 0 and t = 12)

The Functional Recovery Score (FRS) is used to assess restoration of function after discharge.

The eleven-item questionnaire is comprised of three main components: basic activities of daily living (BADL) assessed by four items, instrumental activities of daily living (IADL) assessed by six items, and mobility assessed by one item. Basic activities of daily living comprise 44 percent of the score; instrumental activities of daily living comprise 23 percent, and mobility comprises 33 percent. Complete independence in basic and instrumental activities of daily living and mobility results in a score of 100 percent [20]. Participants will receive instructions from the research assistants on how to fill in the questionnaire. They will receive the questionnaire in advance of the visits at t = 0 and t = 12. At the visits the finalised questionnaire will be inspected and ambiguous entries clarified.

### Organisation

The primary investigator is responsible for the informed consent procedure, final participants' selection, measurements, analysis and reports. The primary investigator will be assisted by two research assistants and two RDs. Data flow will be controlled by the primary investigator. Data-entry and control will be conducted by the research assistants under supervision of the investigator. The primary investigator is responsible for the data cleaning and analysis.

### Statistical analysis

All statistical analysis will be performed using SPSS for Windows. Data will be entered in EXCEL and will subsequently be exported into SPSS software for analysis. Primary analysis for this study will be undertaken using intention to threat principles. 95% confidence intervals will be calculated for the differences in percentages, and medians. Independent samples t-test, Mann-Whitney U test and Chi-square test of association will be used as appropriate to compare groups at baseline. Ceiling and floor effect will be taken into account in the analysis of the questionnaires. In order to test the independent contribution of the intervention on the outcome variables, multivariate regression analysis will be used to adjust for the possible confounders. Specifically, the concordance between the GPs knowledge of the medical treatment and what the participant is actually taken plus the degree to which the GP have implemented the recommended follow-up as described in the hospital discharge letter, in respectively, the intervention and control group, will be used.

The analysis will be undertaken by the principal investigator blinded to the randomisation.

### Ethics

The protocol has been send to the Danish Ethical Board which has concluded that approval is not needed and that the project can be carried on as described.

## Discussion

This project is the first to combine individualised nutritional intervention with intervention from GPs.

We have chosen not to use strict exclusion criteria, but to include all eligible patients even though they are suffering from a variety of (chronic) diseases. Their homogeneity stems from their age (65+ years old), nutritional risk and background of disease (non-surgical). If the results of a broad study like this one are positive, it justifies wide implementation, because the included group is representative for a mixed elderly population; in contrast, selection of a more specific group would make the intervention less applicable to other patients group.

In Denmark it is recommended that the nutritional management of (geriatric) patients involves the provision of high energy, high protein diets and individualised nutritional therapy [15], however the evidence for this, is limited. A review of the literature highlighted that most nutrition support provided for geriatric patients is based on the provision of a standard volume of commercial oral nutritional supplements rather than individualised therapy [21]. Specifically, former nutritional intervention studies among geriatric medical patients after discharge has all used commercial oral nutritional supplements [5-8].

A comprehensive approach to nutritional therapy combining individual education, motivation and counselling, dietary modification and supplementation offered by a RD, differs from previous work, however was deemed necessary given the limited evidence that commercial oral supplements alone can improve outcomes in this frail group.

Strength training is an effective intervention for improving physical functioning in older people [22]. In Denmark it is part of the legislation to offer some patients rehabilitation in the form of physiotherapy. In this study, it was therefore decided not to include training as a specific part of the comprehensive intervention, but instead register if there is a difference in the prevalence between the two groups.

Most of the former discharge studies may have had an intervention time that was too short to have a realistic chance of detecting differences in morbidity, functional status or quality of life. According to a recent Cochrane Review future trials need to have sufficient statistical power and length of follow-up to be able to detect any beneficial effects [23]. The follow-up period of 12 weeks is therefore chosen because this seems a reasonable time to achieve benefits of nutritional intervention in older people at nutritional risk. Further, in a former study of discharge follow-up in patients home by GPs, there was seen a significant different in the number of re-admissions after 12 weeks [12].

### Weaknesses

In the Danish study offering discharge follow-up in patients' home [12] the inclusion criteria were aged 78+ years. This means that the participants in this study will be younger, probably less frail and maybe less susceptible to re-admissions. However in the former nutritional intervention studies among 65+ year old geriatric patients, at risk of or already undernourished, the prevalence of re-admissions has been high - up till 56% [5].

Also, in the Danish study offering discharge follow-up in patients home, district nurses were part of the intervention [12]. Due to structural changes in the involved municipalities this is no longer possible. To try to compensate for this, regularly contact with district nurses will be arranged.

In this study there are possibilities of contamination between intervention and control groups since some GPs may be involved in both groups. Since the aim of the study can not be blinded to the GPs, the chosen method may raise the GPs attention in relation to nutritional aspects in both intervention and control participants.

On the other hand the GPs will be paid by the project for their discharge contacts, since such contacts are not yet obligatory. This fact may bias this study towards a *better *effect than can be obtained in daily practice.

## Conclusions

It is important to provide adequate rehabilitation after hospitalisation to rehabilitate people as close to pre-morbid function as possible so that physical decline, hospital re-admission and even nursing home admission are avoided. The result of this project will hopefully help to guide the development of more effective rehabilitation programs following hospital admissions, which may ultimately lead to reduced health care costs, and improvement in mobility, independence and quality of life for geriatric patients at nutritional risk.

## Competing interests

The authors declare that they have no competing interests.

## Authors' contributions

All authors contributed to the design of the study. AMB prepared the grant application and drafted the manuscript, while SK, BSH, RLS and KTJ contributed to drafts of the manuscript. All authors have read and approve the publication of the final manuscript.

## Pre-publication history

The pre-publication history for this paper can be accessed here:

http://www.biomedcentral.com/1471-2318/11/90/prepub
